# Supplementary material for: Distinct submembrane localisation compartmentalises cardiac NPR1 and NPR2 signalling to cGMP
Source: Nat Commun. 2018 Jun 22;9:2446. doi: 10.1038/s41467-018-04891-5 (PMC6014982; doi:10.1038/s41467-018-04891-5)
Supplement: Supplementary file 1 — Supplementary Information [file 41467_2018_4891_MOESM1_ESM.pdf]

# SUPPLEMENTARY INFORMATION

# Distinct submembrane localisation compartmentalises cardiac NPR1 and NPR2 signalling to cGMP

Subramanian et al.

Supplementary Figures S1-S13
Supplementary Tables S1-S3
Supplementary Reference

## Supplementary Figures

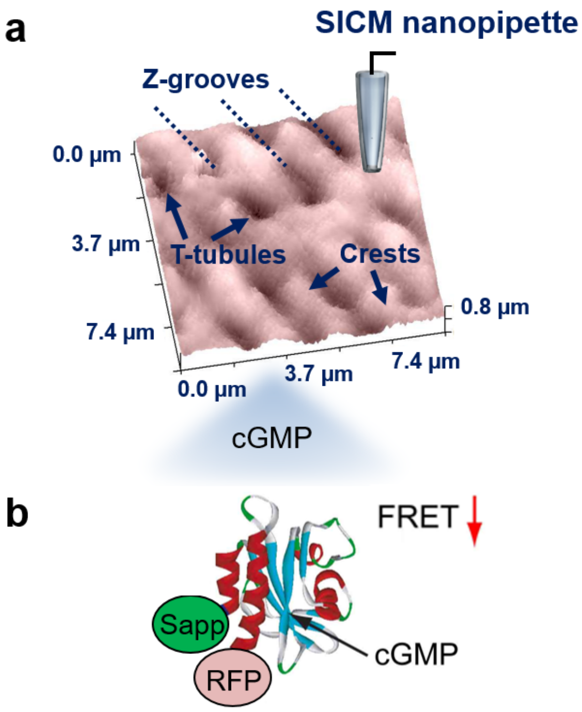


**Supplementary Figure 1**. **Schematic diagram of the SICM/FRET measurements. a**, Firstly, we obtained a 10x10 µm scan of a living mouse VM cell surface using hopping probe SICM. Next, the scanning nanopipette was precisely positioned over the area of interest (T-tubular opening or cell crest) before local ligand application with simultaneous FRET recording in the cell cytosol (from a region spanning the whole cell). Under the used application pressure of 276 kPa (40 psi), the calculated delivered amount of the ligand through the 100 MΩ nanopipette was 1.1 pL/s, which corresponds to ~130-200 pL volume applied during the course of a typical 2-3 min long stimulation[18]. In this case, the ligand originally present in the pipette is diluted >1:100 fold before reaching the membrane. When 100 µM of NPs was filled into the pipette, the actual ligand concentrations at individual receptors were well below 1 µM (see also Supplementary Fig. 2). **b**, cGMP was visualised using the transgenically expressed cytosolic cGMP biosensor red cGES-DE5. This sensor contains a cGMP binding domain from PDE5 sandwiched between T-sapphire (Sapp) and Dimer2 (RFP) fluorescent proteins and shows a decrease of FRET upon cGMP binding.

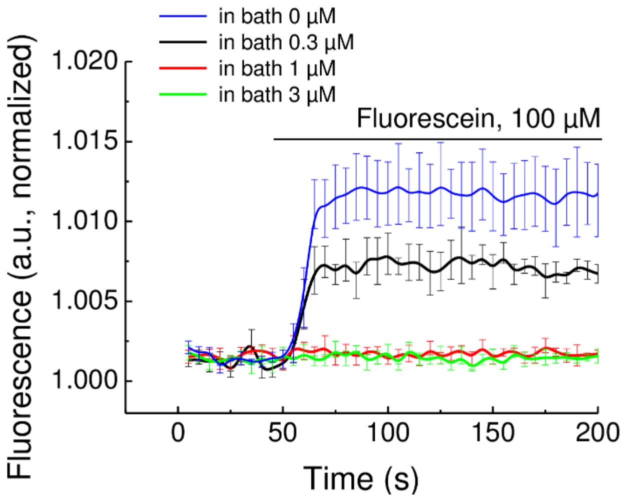


**Supplementary Figure 2**. **Measurement of ligand dilution during application from a SICM nanopipette.** 100 µM fluorescein solution was loaded into pipette and applied under the same experimental conditions as described above onto the membrane of wildtype VMs, while measuring fluorescence in the same focal plane and having different amounts of fluorescein in the bath solution. Signals disappear when bath fluorescein concentration has reached 1 µM, suggesting a ~1:100 ligand dilution during SICM-based pressure application. Averaged fluorescence time lapse traces are shown with individual data points representing means ± s.e.m. values (n=4-5) each.

a

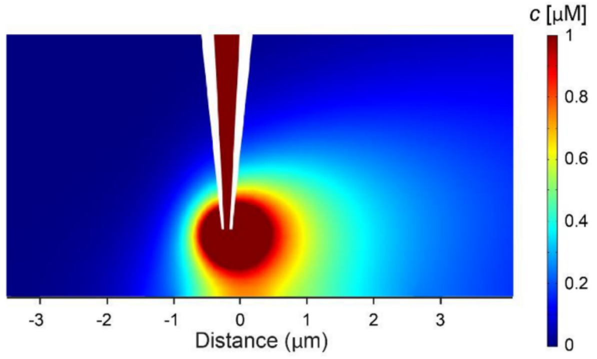


b

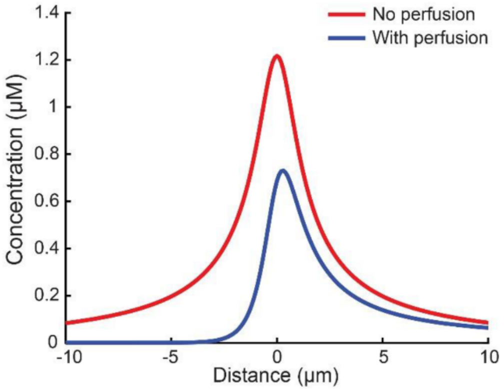


**Supplementary Figure 3**. **Finite element simulations of NP concentrations delivered to the membrane using a SICM nanopipette. a,** Concentration profile over the cell surface during ligand application under standard experimental conditions from a pipette filled with 100 µM NP, positioned at 1 µm distance from the surface and with perfusion along the *x*-direction. The maximum concentration at the cell surface is ~700 nM. **b**, Graphical representation of the concentration profiles obtained with and without perfusion.

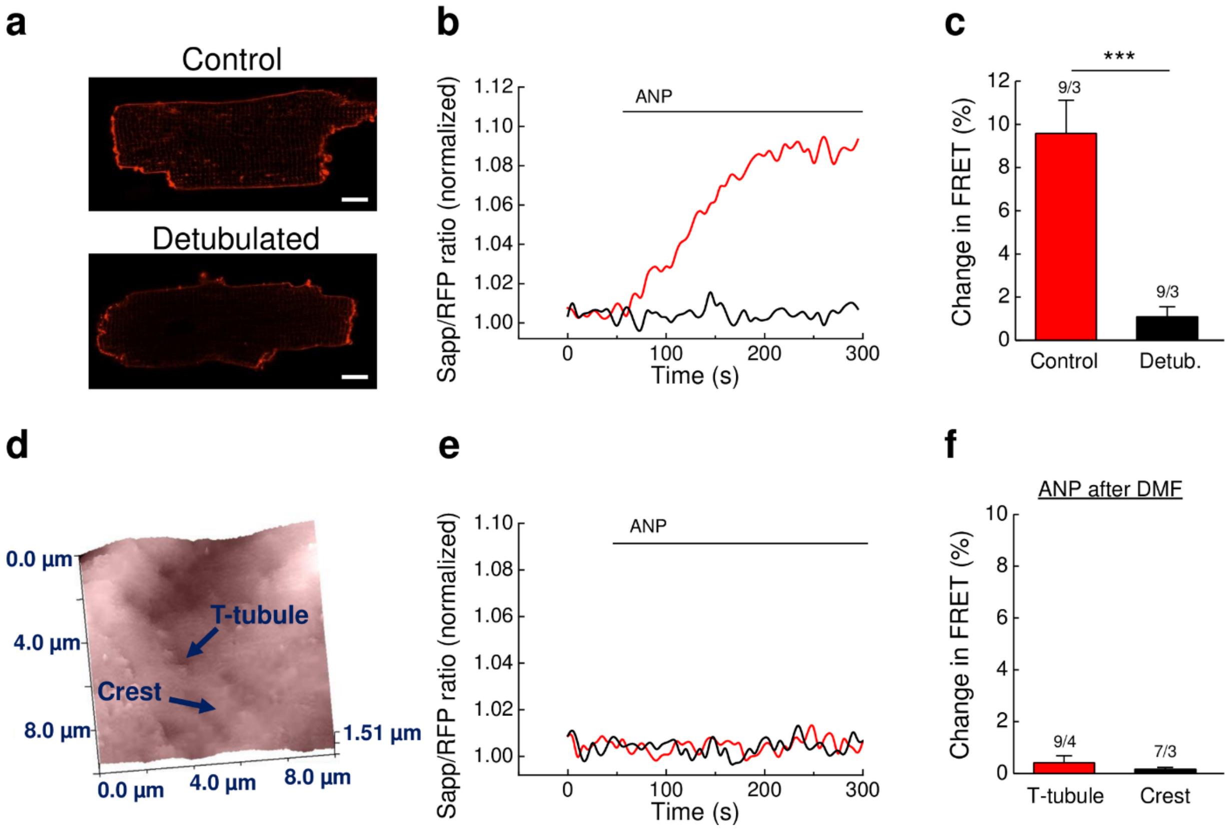


**Supplementary Figure 4. Responses to ANP in cells subjected to dimethylformamide induced detubulation** (DMF, 1.5 M in buffer A for 15 min at room temperature). **a,** Confocal images of control di-8-ANEPPS live staining (100 µM for 10 min in buffer A) showing a clear loss of intracellular T-tubules. **b**, Representative FRET traces from normal and detubulated cells treated with 100 nM ANP globally applied to the bath solution. Data analysis is in c. Representative scan (**d**), traces (**e**) and analysis (**f**) of SICM/FRET recordings in detubulated cells treated with ANP as described in Fig. 1. Data represent means ± s.e.m. values from the indicated number of cells/mice. *** p<0.001 by mixed ANOVA followed by Wald $\chi^2$-test.

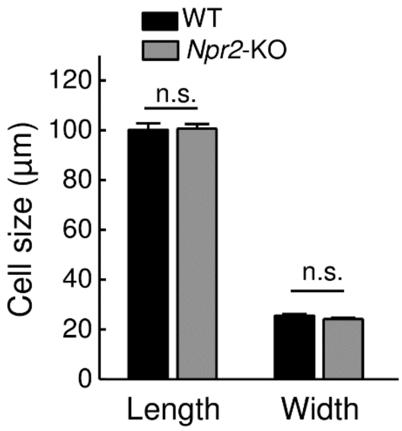


**Supplementary Figure 5**. **Cell size analysis of wildtype vs. *Npr2* knockout VMs** shows no significant differences in cell length and width. These parameters were measured using bright field cell images and ImageJ software. Data are means ± s.e.m., n=47 and 37 measured cells, respectively. n.s., not significant by mixed ANOVA followed by Wald $\chi^2$-test.

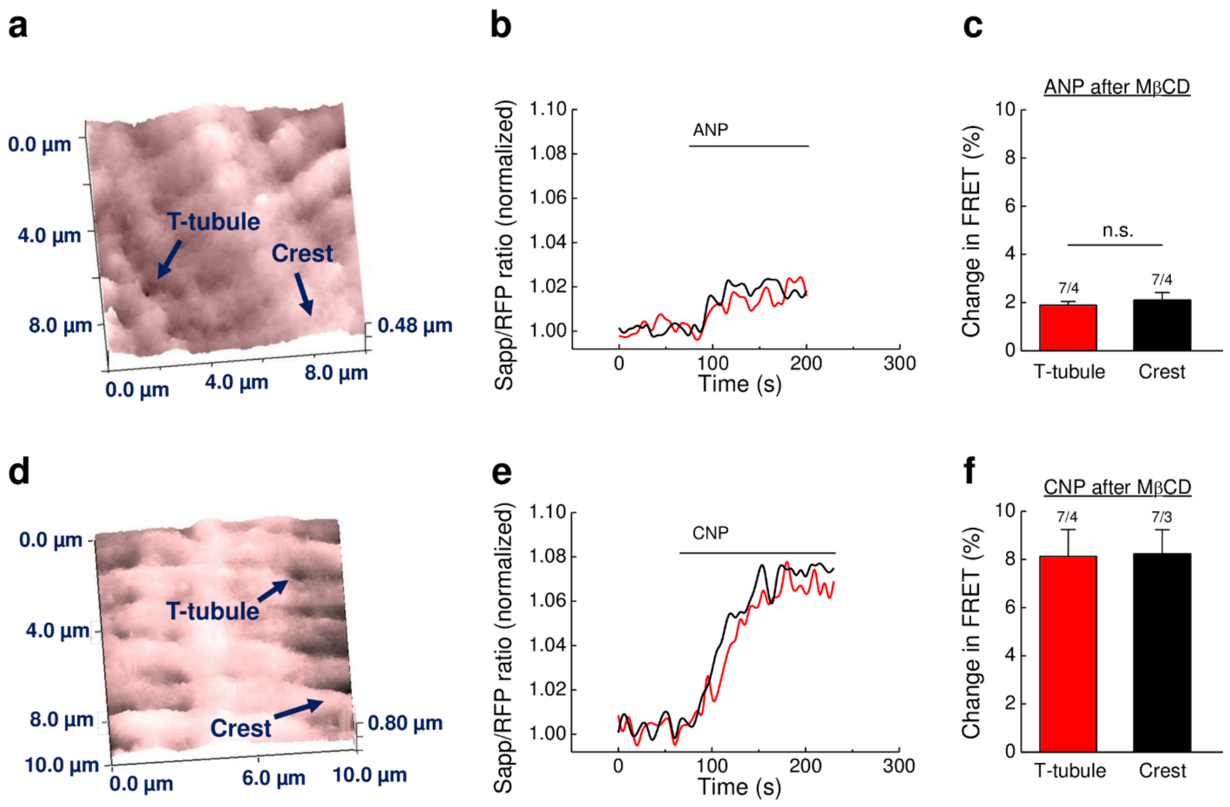


**Supplementary Figure 6**. **SICM/FRET experiments performed after VM incubation with the cholesterol depleting agent methyl-β-cyclodextrin** (MβCD, 1 mM, 1h). Representative scan (**a**) **traces (b)**, and data quantification (**c**) for the effect of ANP on the crest vs, T-tubular stimulation. Cholesterol depletion leads to NPR1 redistribution from the T-tubules to cell crests. **d-f**, effect of CNP is not significantly affected by MβCD treatment. Data represent means ± s.e.m. values from the indicated number of cells/mice. n.s., not significant by mixed ANOVA followed by Wald $\chi^2$-test.

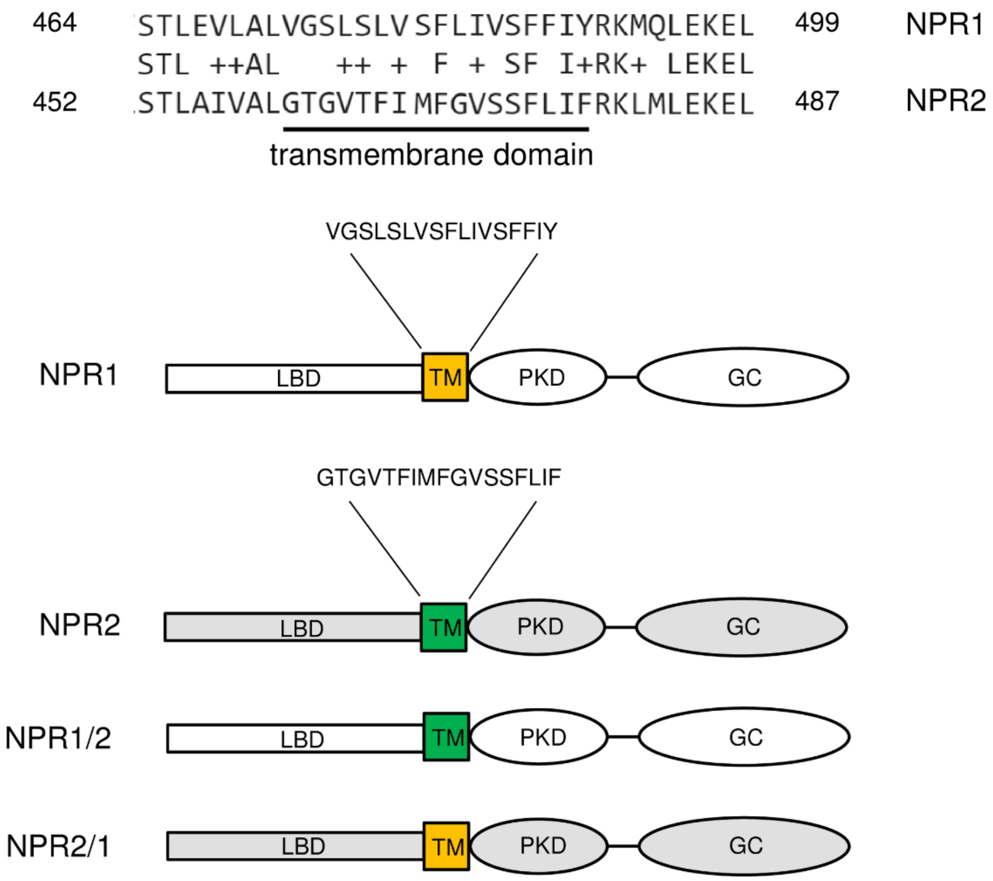


**Supplementary Figure 7. NPR1 and NPR2 transmembrane (TM) domain protein sequence alignment and schematic representation of the chimeric constructs used for FRAP analysis.** Both receptors show relatively low homology in transmembrane domain sequence. TM domain of NPR1 is in yellow, TM domain of NPR2 is in green. NPR1/2 chimeric construct represents NPR1 with TM domain derived from NPR2. Conversely, NPR2/1 is NPR2 with TM domain from NPR1. All constructs were fused to enhanced yellow fluorescent protein (EYFP) sequence on their C-termini. LBD, ligand-binding domain; TM, transmembrane domain; PKD, protein kinase-like domain; GC, guanylate cyclase domain.

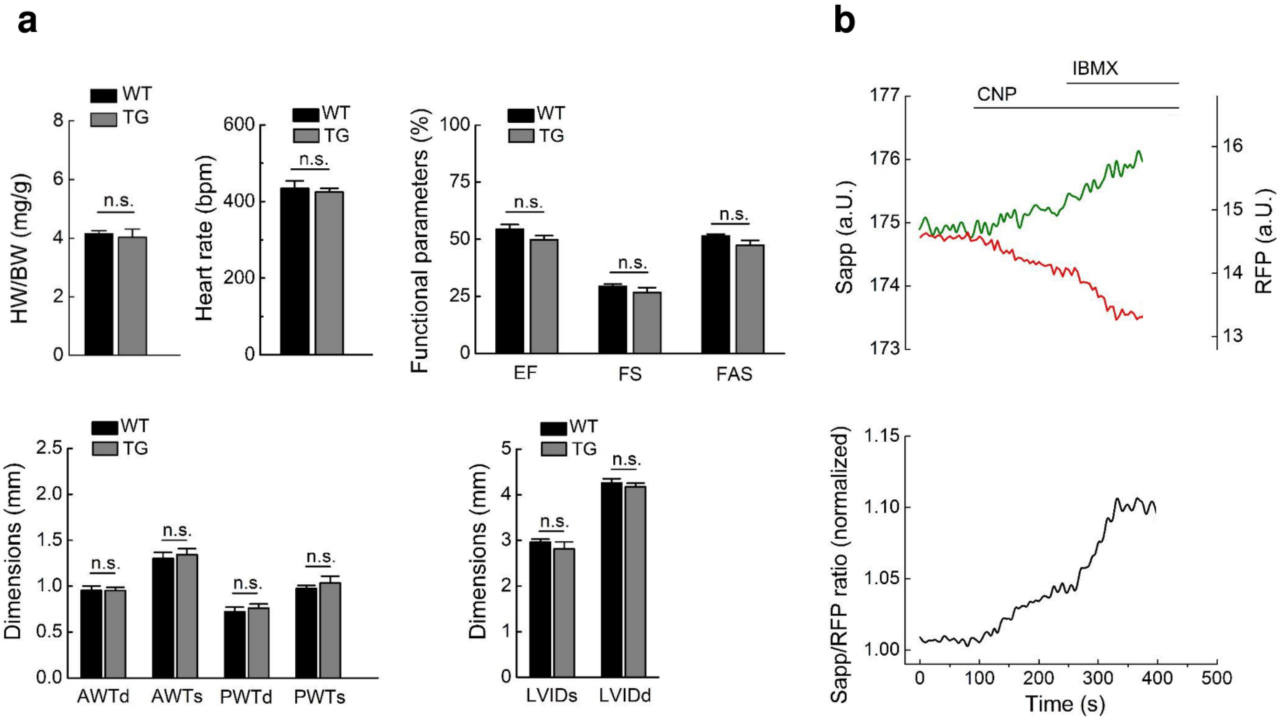


**Supplementary Figure 8**. **Characterisation of the pmDE5 transgenic mice**. **a**, Echocardiography of the transgenic (TG, n=11) mice compared to wildtype (WT, n=10) littermates did not reveal any abnormal heart dimensions, altered heart rate or contractile dysfunction. AWTd, anterior wall thickness in diastole; AWTs, anterior wall thickness in systole; BW, body weight; EF, ejection fraction; FAS, fractional area shortening; FS, fractional shortening; HW, heart weight; LVIDd, left ventricular end-diastolic dimension; LVIDs, left ventricular end-systolic dimension; PWTd, posterior wall thickness in diastole; PWTs, posterior wall thickness in systole. All differences between WT and TG are not significant (n.s.) by one-way ANOVA. **b**, FRET measurements in isolated pmDE5 transgenic CMs show clear decreases of FRET (represented as an increase in donor/acceptor ratio) upon stimulation with CNP (100 nM) and IBMX (100 μM). Representative trace of 10 experiments.

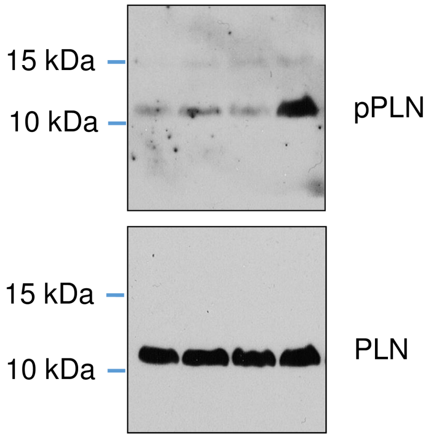


**Supplementary Figure 9**. **Uncropped blots for the gels shown in Figure 6c.**

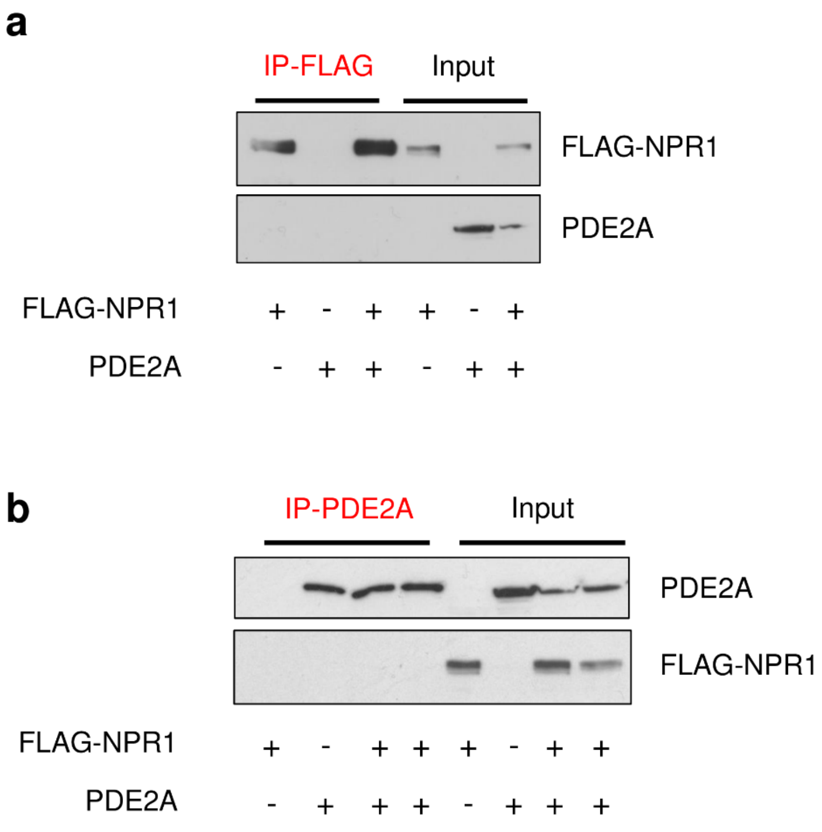


**Supplementary Figure 10**. **Representative co-immunoprecipitation experiments** (n=3 each) performed with lysates from HEK293A cells transfected with FLAG-NPR1 and/or PDE2A plasmids as indicated below the immunoblots. No direct co-immunoprecipitation between NPR1 and PDE2A can be observed in any direction.

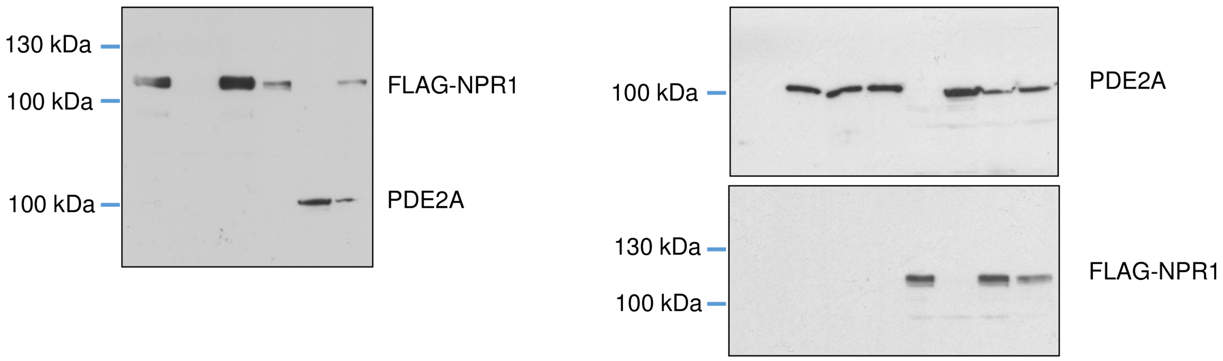


**Supplementary Figure 11**. **Uncropped blots for the gels shown in Supplementary Figure 10.**

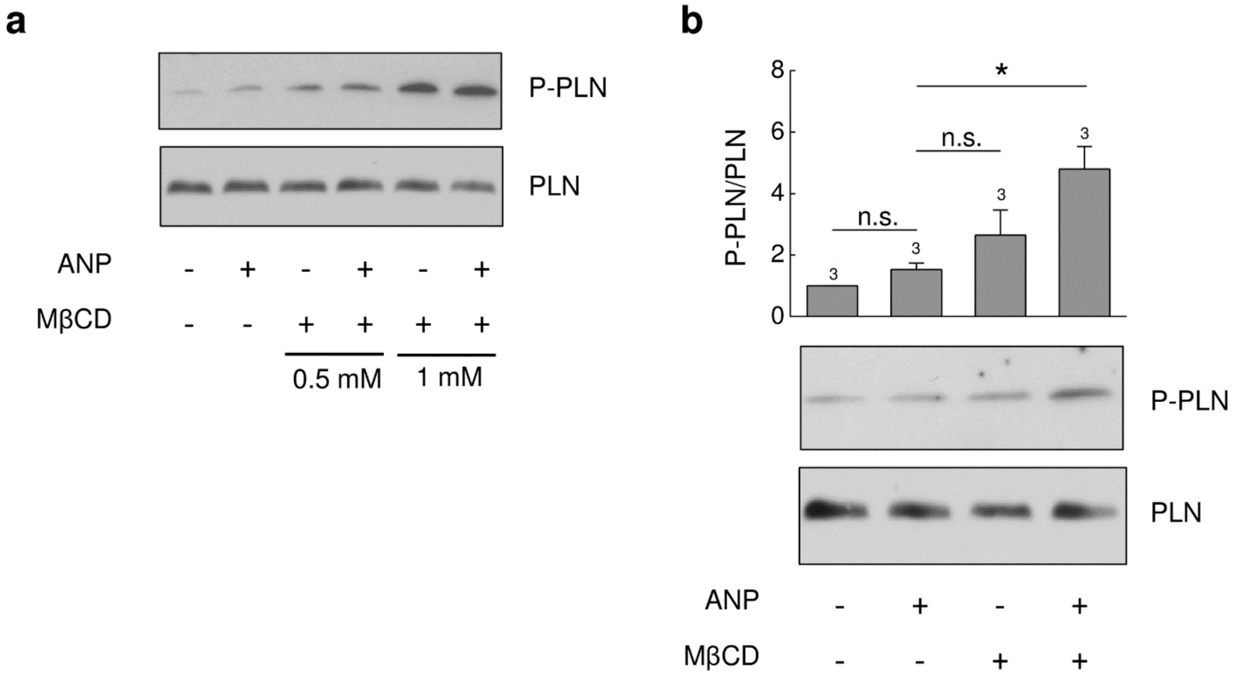


**Supplementary Figure 12**. **Phospholamban (PLN) phosphorylation on Ser16 in VMs preincubated with MβCD** (for 45 min at 37°C). **a**, MβCD alone causes PLN phosphorylation which is especially pronounced at 1 but not at 0.5 mM. **b**, After preincubation with 0.5 mM MβCD or with vehicle, cells were stimulated using 100 nM ANP for 10 min and lysed for immunoblot analysis. Preincubation with 0.5 mM MβCD leads to ANP-induced PLN phosphorylation. Representative immunoblots (n=3). * P<0.05, n.s. - not significant by one-way ANOVA.

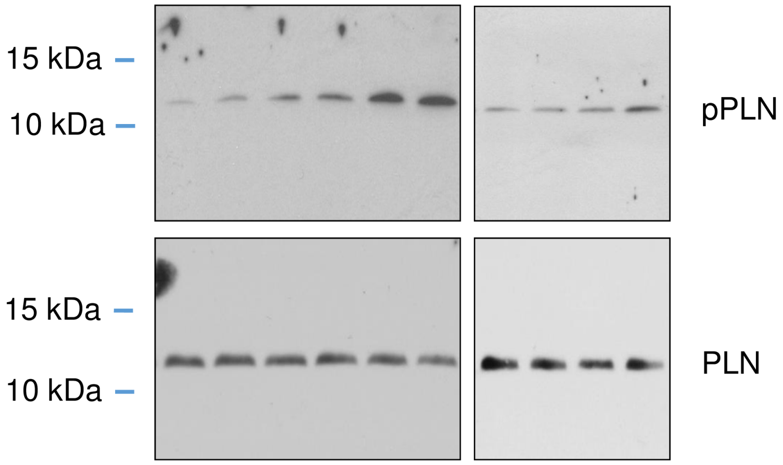


**Supplementary Figure 13. Uncropped blots for the gels shown in Supplementary Figure 12.**

## Supplementary Tables

**Supplementary Table 1.** List of primers used in the study.

| **Primers for genotyping** | |
|---|---|
| MHC Forward | TGACAGACAGATCCCTCCTAT |
| YFP Reverse | GGATGCTCAGGTAGTGGTT |
| **Primers for plasmid construction** | |
| NPR2 forward | AAAAGCTTATGGCACTGCCATCCC |
| NPR2 reverse | AAAGAATTCGCAGGAGTCCGGGAGG |
| NPR1 forward | AAAGCTAGCGCCATGCCGGGCTCC |
| NPR1 reverse | AAACTCGAGTCCCCTAGTGCTACATCCCCGCT |
| NPR1 TMD-Forw | GTGGCCCTGGGCACGGGAGTCACCTTCATCATGTTTGGTGTTTCCA GTTTCCTAATTTTCAGGAAGATGCAGCTGGAA |
| NPR1 TMD-Rev | GAAAATTAGGAAACTGGAAACACCAAACATGATGAAGGTGACTC CCGTGCCCAGGGCCACCTCCAGTGTGGAAAAGTGG |
| NPR2 TMD-Forw | GTGGGCAGCCTCTCTCTGATTAGCTTTCTGATTGTGTCTTTCTTCAT ATACCGGAAGCTGATGCTGGA |
| NPR2 TMD-Rev | GTATATGAAGAAAGACACAATCAGAAAGCTAATCAGAGAGAGGC TGCCCACCAGGGCCACGATTGCC |

**Supplementary Table 2.** Parameter values used in the finite element simulations.

| **Name** | **Description** | **Value** |
|---|---|---|
| $R_0$ | Inner tip radius | 40 nm |
| $R_1$ | Outer tip radius | 80 nm |
| $\theta$ | Inner half-cone angle | 3° |
| $h$ | Pipette-surface distance | 1 μm |
| $D$ | ANP/CNP diffusivity[a] | $3\times10^{-10}$ $m^2/s$ |
| $c_0$ | ANP/CNP pipette conc. | 100 μM |
| $\Delta p$ | Applied pressure | 6.8 kPa |
| $v_{perf}$ | Perfusion velocity[b] | 1 mm/s @ $z$ = 1 μm |

[a] Estimated from Eq. 5 in Hosoya et al.[1] with MW(ANP) = 3.1 kDa and MW(CNP) = 2.2 kDa.
[b] Measured by tracking suspended particles in the perfusion setup. The perfusion velocity is assumed to vary linearly with distance from the surface in the simulations (zero velocity at the surface).

**Supplementary Table 3.** Boundary conditions for the perfusion simulations.

| | **Boundary condition** |
|---|---|
| **Creeping flow – delivery** | |
| *x* = -30 µm; *x* = 40 µm; *y* = -30 µm; *z* = 30 µm | ***Outlet:*** *p* = 0 |
| Top of the pipette | ***Inlet:*** *p* = 6.8 kPa |
| *y* = 0 µm | ***Symmetry*** |
| All other boundaries | ***Wall – no slip*** |
| **Creeping flow – perfusion**[a] | |
| *x* = -30 µm; *x* = 40 µm; *y* = -30 µm | ***Outlet:*** x-velocity = 1[mm/s]×*z*/(1[µm]) |
| *z* = 30 µm | ***Open boundary:*** Normal stress = 0 |
| *y* = 0 µm | ***Symmetry*** |
| All other boundaries | ***Wall – no slip*** |

## Supplementary Reference


1. Hosoya, O. *et al.* Determination of diffusion coefficients of peptides and prediction of permeability through a porous membrane. *J Pharm Pharmacol* **56**, 1501-1507 (2004).
